# Baicalein Attenuates High Glucose and Sodium Palmitate-Induced Ferroptosis in Cardiomyocytes via the Nrf2/SLC7A11/GPX4 Signaling Pathway

**DOI:** 10.3390/ijms27146391

**Published:** 2026-07-18

**Authors:** Huan Wang, Yuhan Xu, Lei Wang, Wanting Meng, Yanwu Xu, Haidong Guo

**Affiliations:** 1 School of Integrative Medicine, Shanghai University of Traditional Chinese Medicine, Shanghai 201203, China; huanwangshutcm@outlook.com (H.W.); wongleeedu@163.com (L.W.); mwting0220@163.com (W.M.); 2 Department of Electrical and Computer Engineering, P.C. Rossin College of Engineering and Applied Science, Lehigh University, Bethlehem, PA 18015, USA; yux725@lehigh.edu

**Keywords:** diabetic cardiomyopathy, high glucose, sodium palmitate, baicalein, cardiomyocyte, ferroptosis, network pharmacology

## Abstract

Diabetes mellitus (DM) is increasing rapidly worldwide, and diabetic cardiomyopathy (DCM) has become a leading cause of death in diabetic patients. Therefore, effective strategies for the prevention and treatment of DCM are urgently needed. Ferroptosis, a form of regulated cell death, has been implicated in the pathogenesis of DCM. This study integrated network pharmacology, data mining, molecular docking, molecular dynamics simulations, and in vitro experiments to investigate whether baicalein inhibits high glucose and sodium palmitate (HG + PA)-induced ferroptosis in cardiomyocytes and to elucidate the underlying mechanisms. Baicalein significantly improved the viability of H9c2 and AC16 cells, reduced cell death, and decreased LDH release under HG + PA conditions. Network pharmacology predicted that the protective effects of baicalein against HG + PA-induced cardiomyocyte injury were associated with ferroptosis regulation. Transcriptomic data mining further identified ferroptosis-related pathway enrichment in complementary in vitro and diabetic rat cardiac datasets. Molecular docking predicted favorable binding poses of baicalein with Nrf2, SLC7A11, and GPX4, while molecular dynamics simulations suggested general stability of the modeled complexes. In vitro experiments further confirmed ferroptosis involvement, as the ferroptosis inhibitor Ferrostatin-1 (Fer-1) reversed the HG + PA-induced decline in cell viability. Conversely, the ferroptosis inducer Erastin diminished cell survival and antagonized the protection conferred by baicalein, indicating that baicalein acts by inhibiting ferroptosis. Baicalein reduced lipid peroxidation, MDA and Fe^2+^ levels, and the mRNA expression of *ACSL4* and *PTGS2*, while restoring the GSH/GSSG ratio and the protein expression of Nrf2, SLC7A11, and GPX4. These protective effects were partially reversed by the Nrf2 inhibitor ML385. In conclusion, baicalein protects cardiomyocytes from HG + PA-induced injury by activating the Nrf2/SLC7A11/GPX4 signaling pathway and inhibiting ferroptosis.

## 1. Introduction

The prevalence of diabetes mellitus (DM) has increased markedly. The global number of adults with DM is projected to increase from 463 million (9.3%) in 2019 to 700 million (10.9%) by 2045 [1]. Cardiovascular complications are a major cause of morbidity and mortality in patients with DM [2]. Diabetic cardiomyopathy (DCM), first described in 1972 [3], is defined as diabetes-induced structural and functional impairment of the myocardium in the absence of coronary artery disease, hypertension, valvular heart disease, or other primary cardiac disorders [4]. DCM is often clinically silent at an early stage [5] but is commonly associated with left ventricular hypertrophy and diastolic dysfunction, and may gradually progress to systolic dysfunction, arrhythmia, heart failure, and even sudden cardiac death [6,7,8]. Current management mainly relies on glycemic control and symptomatic treatment [9], whereas therapeutic strategies directly targeting diabetes-induced myocardial injury remain limited [10]. Therefore, identifying effective agents and molecular targets for DCM-related cardiomyocyte injury is of considerable clinical importance.

DCM develops through a network of metabolic and molecular disturbances [11], including glucotoxicity, lipotoxicity [12], impaired insulin signaling [13], mitochondrial dysfunction [14], oxidative stress, endoplasmic reticulum stress [15], renin–angiotensin–aldosterone system activation [16], disrupted calcium handling [17], inflammatory responses [18] and regulated cell death [19,20,21,22,23]. Among these mechanisms, cardiomyocyte death plays a central role in the progression of diabetic myocardial injury because terminally differentiated cardiomyocytes have limited regenerative capacity [20]. Thus, defining the specific forms and regulatory mechanisms of cardiomyocyte death may help identify therapeutic targets for DCM [24].

Ferroptosis is an iron-dependent, non-apoptotic form of regulated cell death characterized by excessive lipid peroxidation, intracellular iron accumulation, glutathione depletion, and impaired glutathione peroxidase 4 (GPX4) activity [25,26]. Under diabetic metabolic stress, high glucose and sodium palmitate (HG + PA) exposure can promote reactive oxygen species generation, intracellular iron accumulation, lipid peroxidation, glutathione depletion, and GPX4 dysfunction, thereby driving ferroptotic cardiomyocyte injury [27,28,29,30,31]. Accumulating evidence suggests that ferroptosis contributes to the development of DCM, and pharmacological inhibition of ferroptosis may provide a promising therapeutic strategy for diabetic myocardial injury [26,32]. However, the upstream mechanisms governing ferroptosis under diabetic metabolic stress and the relevant therapeutic targets remain incompletely understood.

Given the complex pathogenesis and involvement of multiple targets in DCM, bioactive compounds derived from traditional Chinese medicine have emerged as potential therapeutic candidates owing to their multitarget effects on oxidative stress, inflammation, mitochondrial dysfunction, fibrosis, and regulated cell death [33]. Baicalein, a major flavonoid compound isolated from the roots of *Scutellaria baicalensis Georgi* and previously identified as a natural ferroptosis inhibitor [34], has been reported to exert antioxidant, anti-inflammatory, metabolic regulatory, and cardioprotective effects [35,36,37,38]. Previous studies have reported that baicalein suppresses ferroptosis in cardiomyocytes exposed to ischemia/reperfusion injury, inflammatory stimulation, or lipotoxic stress, suggesting a potential anti-ferroptotic role in cardiomyocyte injury [39,40,41]. However, whether baicalein protects cardiomyocytes against combined HG + PA-induced glucolipotoxic injury by inhibiting ferroptosis remains unclear.

The Nrf2/SLC7A11/GPX4 signaling pathway is a key endogenous defense system against oxidative stress and ferroptosis [42,43,44]. Nrf2 functions as an upstream transcriptional regulator of antioxidant and anti-ferroptotic responses, partly by regulating SLC7A11-mediated cystine uptake and glutathione biosynthesis, which in turn supports GPX4-dependent detoxification of lipid peroxides and limits ferroptotic cell death [45,46,47]. For example, exogenous H_2_S cardiomyocytes was reported to reduce high glucose/palmitate-induced ferroptosis in HL-1 cardiomyocytes and cardiac ferroptosis in db/db diabetic mice by activating the Nrf2/GPX4/GSH pathway [48]. Irisin inhibited palmitic acid-induced ferroptosis in H9c2 rat cardiomyocytes and AC16 human cardiomyocytes through the System Xc^−^/GSH/GPX4 axis [31]. Similarly, muscone attenuated hypoxia-induced ferroptosis in H9c2 cardiomyocytes and myocardial ferroptosis in a rat myocardial infarction model via the Nrf2/System Xc^−^/GPX4 signaling pathway [44]. Therefore, modulation of the Nrf2/SLC7A11/GPX4 axis may represent an important mechanism for protecting cardiomyocytes from HG + PA-induced metabolic injury. In this study, we established in vitro models of DCM-like cardiomyocyte injury using AC16 and H9c2 cells exposed to HG + PA conditions, and integrated network pharmacology, data mining, molecular docking, molecular dynamics simulations, and pharmacological intervention to explore the protective effects and underlying mechanisms of baicalein. Specifically, we aimed to determine whether baicalein alleviates HG + PA-induced myocardial cell injury by modulating the Nrf2/SLC7A11/GPX4 pathway and inhibiting ferroptosis. These findings may provide experimental evidence for the potential application of baicalein in DCM-associated cardiomyocyte injury.

## 2. Results

### 2.1. Establishment of HG + PA-Induced Cardiomyocyte Injury Models and Optimization of Baicalein Treatment Concentrations

**Figure 1:**
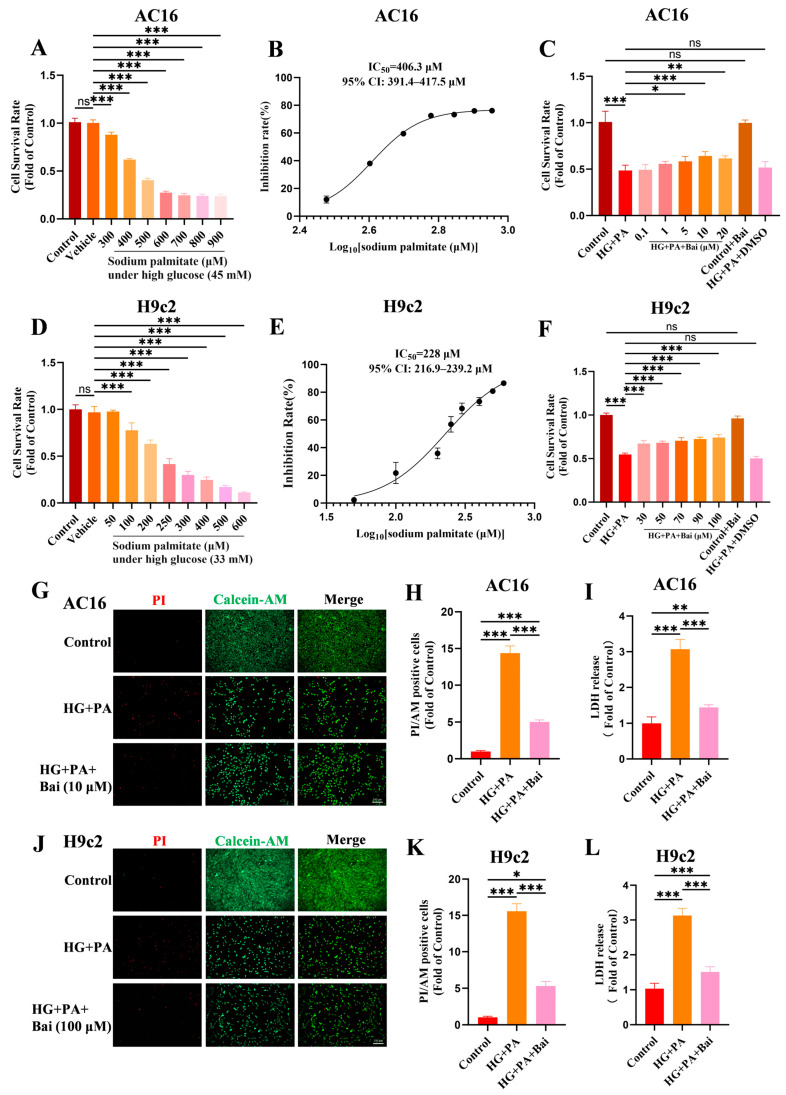
Establishment of HG + PA-induced cardiomyocyte injury models and screening of the optimal working concentration of baicalein (Bai). (**A**,**D**) Cell viability of AC16 and H9c2 cells exposed to increasing concentrations of sodium palmitate (PA) under high-glucose (HG) conditions (n = 6). (**B**,**E**) Dose–response curves and calculated IC_50_ values for PA-induced cytotoxicity in AC16 and H9c2 cells (n = 6). (**C**,**F**) Effects of different concentrations of baicalein on the viability of HG + PA-treated cardiomyocytes (n = 6). (**G**,**J**) Representative images of Calcein-AM/PI staining in AC16 and H9c2 cells (live cells/green, dead cells/red; scale bar = 100 μm) (n = 3). (**H**,**K**) Quantification of PI-positive cells (n = 3). (**I**,**L**) LDH release assay showing the effects of baicalein on HG + PA-induced cytotoxicity in AC16 and H9c2 cells (n = 6). Data are presented as the mean ± SD. * *p* < 0.05; ** *p* < 0.01; *** *p* < 0.001; ns, not significant.

AC16 human cardiomyocytes and H9c2 rat cardiomyoblasts were used to establish in vitro models of HG + PA-induced cardiac cell injury [49,50]. In the presence of 45 mM glucose for AC16 cells and 33 mM glucose for H9c2 cells, increasing concentrations of sodium palmitate (PA) reduced cell viability in a concentration-dependent manner in both cell lines (Figure 1A,D). Dose–response analysis showed that the half-maximal inhibitory concentration (IC_50_) of PA was 406.3 μM in AC16 cells, with a 95% confidence interval of 391.4–417.5 μM (Figure 1B), whereas the IC_50_ of PA in H9c2 cells was 228 μM, with a 95% confidence interval of 216.5–239.2 μM (Figure 1E). Based on the respective IC_50_ values, the following HG + PA modeling conditions were used: 45 mM glucose combined with 400 μM PA for AC16 cells and 33 mM glucose combined with 250 μM PA for H9c2 cells.

Next, the working concentration of baicalein was screened under the established HG + PA conditions. Baicalein restored cell viability to varying degrees in HG + PA-treated AC16 and H9c2 cells (Figure 1C,F). Based on the cell viability results, 10 μM baicalein was selected for AC16 cells and 100 μM for H9c2 cells in subsequent experiments.

### 2.2. Baicalein Attenuates HG + PA-Induced Cytotoxicity in AC16 and H9c2 Cells

Calcein-AM/PI staining was performed to assess live/dead cell status after HG + PA treatment. HG + PA markedly increased PI-positive cells in both AC16 and H9c2 cells compared with the control group, whereas baicalein treatment substantially reduced PI-positive staining (Figure 1G,H,J,K), indicating that baicalein alleviated HG + PA-induced cell death.

LDH release was further assessed to evaluate cell injury. Consistent with the Calcein-AM/PI staining results, HG + PA significantly increased LDH release in both AC16 and H9c2 cells, whereas baicalein markedly reduced HG + PA-induced LDH release (Figure 1I,L), indicating that baicalein alleviated HG + PA-induced membrane damage.

Taken together, these results demonstrate that baicalein improves cell viability, reduces cell injury, and attenuates cell death in HG + PA-treated AC16 and H9c2 cells.

### 2.3. Network Pharmacology and Data Mining Identify Ferroptosis Regulation as a Key Mechanism of Baicalein-Mediated Protection

**Figure 2:**
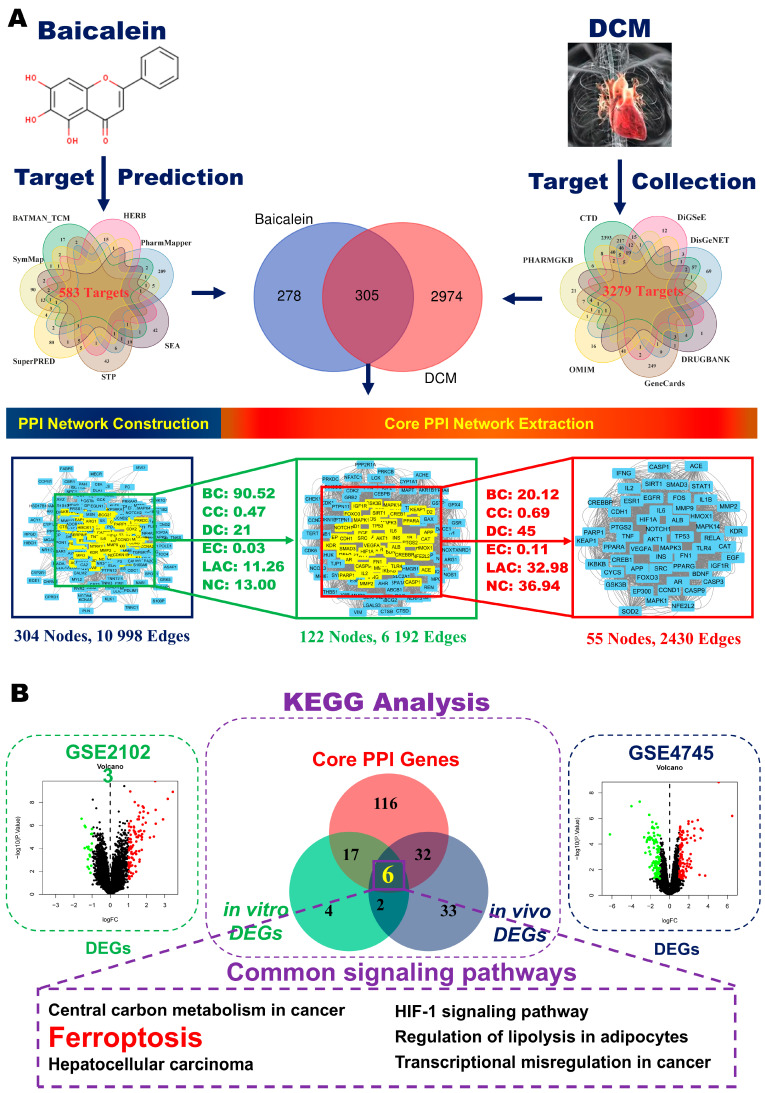
Network pharmacology and data mining analysis of the potential mechanisms underlying baicalein-mediated cardiomyocyte protection. (**A**) Baicalein–DCM candidate target screening and core PPI network construction. (**B**) Integrated KEGG analysis identifies ferroptosis as a common signaling pathway associated with baicalein-mediated cardiomyocyte protection. In the volcano plots, significantly upregulated and downregulated genes are shown in red and green, respectively, whereas genes without significant differential expression are shown in black. All detailed information is provided in Supplementary Materials.

To explore the mechanisms underlying baicalein-mediated protection in diabetic cardiomyopathy (DCM), baicalein-related targets were intersected with DCM-associated genes. After deduplication, 583 baicalein-related targets and 3279 DCM-related genes were obtained from multiple target prediction and disease-gene databases (Tables S1 and S2), yielding 305 overlapping candidate targets for subsequent network and pathway analyses (Figure 2A and Tables S3).

To further prioritize biologically relevant targets for pathway enrichment, a protein–protein interaction (PPI) network was constructed using the overlapping target set. Among the 305 candidate targets, 304 were successfully mapped to the PPI network, generating 10,998 interactions. A two-step topological filtering strategy was then applied to extract highly connected core targets, yielding a core PPI network consisting of 55 nodes and 2430 edges (Figure 2A and Table S4). These core targets were subsequently subjected to KEGG pathway enrichment analysis to identify the major signaling mechanisms associated with baicalein-mediated protection (Table S5).

To strengthen the disease relevance of the predicted pathways, DCM-related transcriptomic datasets were further incorporated. GSE21023 and GSE4745 (Tables S6 and S7) were used as in vitro and in vivo expression datasets, respectively. Differentially expressed genes (DEGs) from these datasets were subjected to KEGG enrichment analysis, and the results were intersected with the KEGG enrichment results of the core PPI network genes. This analysis identified six shared signaling pathways, including ferroptosis, central carbon metabolism in cancer, HIF-1 signaling, regulation of lipolysis in adipocytes, hepatocellular carcinoma, and transcriptional misregulation in cancer (Figure 2B and Table S8). Given its close association with DCM and lipid peroxidation, ferroptosis was prioritized as the key mechanism for subsequent experimental validation.

### 2.4. Pharmacological and In Silico Analyses Implicate the Nrf2–SLC7A11–GPX4 Axis in Baicalein-Mediated Protection Against HG + PA-Induced Ferroptosis

**Figure 3:**
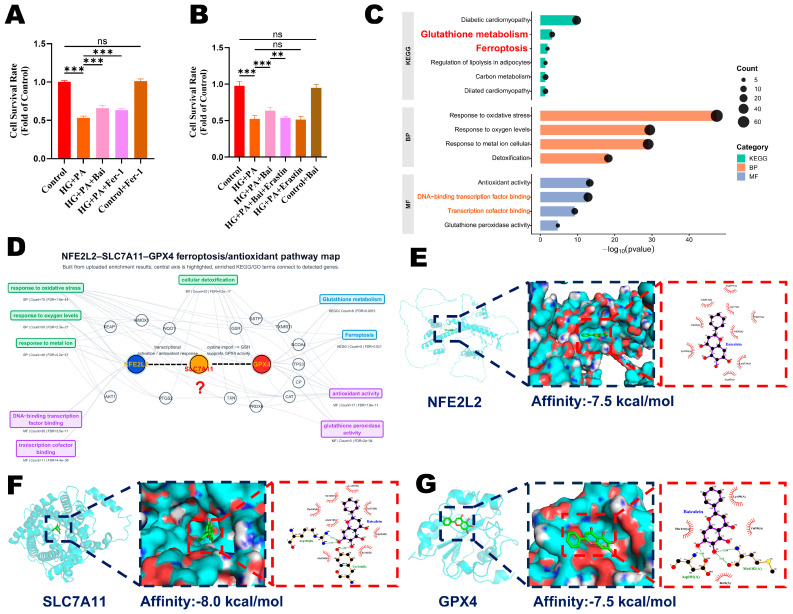
Ferroptosis-focused profiling and docking analysis of baicalein-associated targets in DCM. (**A**) Effects of baicalein and Fer-1 (3 μM) on the viability of HG + PA-treated AC16 cells. (**B**) Effects of Erastin (2 μM) on baicalein-mediated protection in HG + PA-treated AC16 cells. (**C**) GO and KEGG enrichment analyses of drug–disease intersecting targets. (**D**) Core target analysis of ferroptosis-related pathways. (**E**–**G**) Molecular docking of baicalein with NFE2L2, SLC7A11, and GPX4. In the molecular docking panels, baicalein is shown as a green stick model within the protein-binding pocket, whereas the target proteins are displayed as molecular surfaces. The corresponding two-dimensional diagrams illustrate the ligand–protein interaction patterns. Data in A and B are presented as mean ± SD (n = 6). ** *p* < 0.01, *** *p* < 0.001; ns, not significant. Detailed information is provided in Supplementary Materials.

Following the identification of ferroptosis as a candidate pathway, we examined its contribution to HG + PA-induced cytotoxicity. HG + PA exposure markedly reduced AC16 cell viability, whereas baicalein significantly reversed this reduction (Figure 3A). Similarly, the ferroptosis inhibitor Ferrostatin-1 (Fer-1, 3 μM) improved cell viability under HG + PA conditions, suggesting that ferroptosis contributes to HG + PA-induced cytotoxic injury.

Conversely, Erastin (2 μM) markedly weakened the viability-restoring effect of baicalein (Figure 3B), indicating that ferroptosis activation counteracted baicalein-mediated cytoprotection. These findings suggest that baicalein protects AC16 cells against HG + PA-induced cytotoxicity, at least in part, by suppressing ferroptosis.

To ensure comprehensive target coverage, we further performed enrichment analysis of the drug–disease intersecting targets. KEGG analysis showed significant enrichment of pathways related to DCM, glutathione metabolism, and ferroptosis. BP terms were mainly enriched in oxidative stress response, response to oxygen levels, and cellular response to metal ions, while MF terms included antioxidant activity, DNA-binding transcription factor binding, and glutathione peroxidase activity (Figure 3C). Further target–pathway analysis revealed NFE2L2 (Nrf2) and GPX4 as core targets within these ferroptosis-related pathways (Figure 3D and Table S9). Given that the Nrf2–SLC7A11–GPX4 axis is a key intracellular antioxidant pathway that counteracts ferroptosis, these findings suggest that baicalein may attenuate HG + PA-induced cardiomyocyte ferroptosis by targeting key proteins within this axis.

Molecular docking was then performed to assess the potential interactions between baicalein and key proteins within this axis. The analysis predicted energetically favorable binding poses of baicalein with Nrf2, SLC7A11, and GPX4, with docking energies of −7.5, −8.0, and −7.5 kcal/mol, respectively (Figure 3E–G).

### 2.5. Molecular Dynamics Simulations Further Validate the Stability of Predicted Baicalein–Target Complexes

To further validate the docking-derived interaction models, 100-ns molecular dynamics (MD) simulations were performed for baicalein in complex with Nrf2, SLC7A11, and GPX4. Multiple trajectory parameters, including RMSD, RMSF, solvent-accessible surface area (SASA), hydrogen-bond formation, and Gibbs free-energy landscape, were analyzed to evaluate the dynamic stability and interaction behavior of each complex.

**Figure 4:**
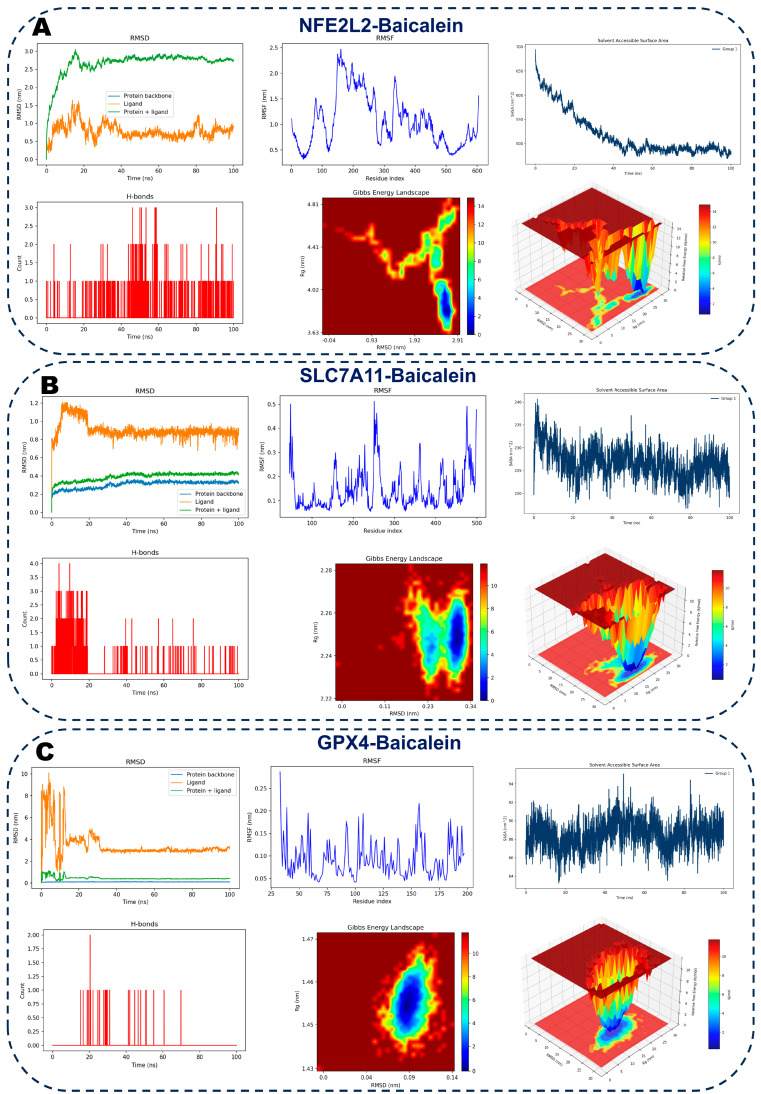
Molecular dynamics simulations of baicalein–target complexes. (**A**–**C**) Molecular dynamics simulation results for NFE2L2–baicalein, SLC7A11–baicalein, and GPX4–baicalein complexes over 100 ns. Each panel shows root mean square deviation (RMSD), root mean square fluctuation (RMSF), solvent-accessible surface area (SASA), hydrogen-bond count, two-dimensional Gibbs free-energy landscape, and three-dimensional free-energy surface.

For the Nrf2–baicalein complex, the RMSD trajectory reached a relatively stable state after initial equilibration, without marked global conformational drift (Figure 4A). RMSF analysis indicated moderate residue-level fluctuations, while SASA and radius-related structural parameters suggested that the overall compactness of the complex was largely maintained. Intermittent hydrogen-bond formation was observed during the simulation, supporting repeated but noncontinuous ligand–protein contacts. The Gibbs free-energy landscape further indicated that the complex sampled relatively stable conformational states.

The SLC7A11–baicalein complex also displayed a stabilized RMSD profile after equilibration, with limited structural fluctuation during the 100-ns simulation (Figure 4B). RMSF and SASA analyses suggested that the protein structure remained dynamically stable, and hydrogen-bond analysis showed transient interactions between baicalein and SLC7A11. Consistently, the Gibbs free-energy landscape supported the presence of energetically favorable conformational states for the predicted complex.

For the GPX4–baicalein complex, the RMSD trajectory remained broadly stable throughout the simulation (Figure 4C). RMSF analysis showed limited local flexibility, and SASA analysis did not reveal major structural expansion or collapse, indicating preserved conformational compactness. Intermittent hydrogen-bond interactions were also detected between baicalein and GPX4, while the Gibbs free-energy landscape further supported the stability of the predicted binding mode.

Together, these MD simulation results suggest that the modeled baicalein complexes with Nrf2, SLC7A11, and GPX4 remained generally stable over the 100-ns simulation period. These simulations provide computational support for the internal stability of the modeled complexes; however, further experimental validation is required to confirm direct binding and cellular target engagement.

### 2.6. ML385 Attenuates Baicalein-Mediated Anti-Ferroptotic Protection by Suppressing the Nrf2–SLC7A11–GPX4 Axis

**Figure 5:**
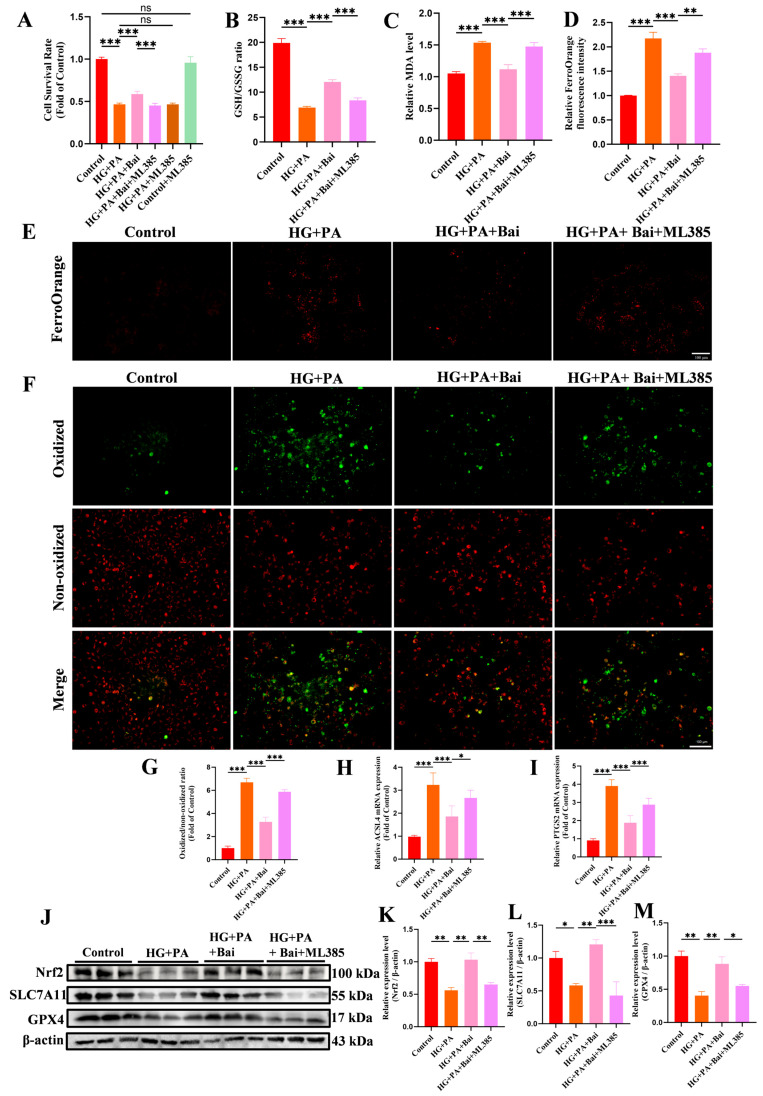
ML385 attenuates baicalein-mediated anti-ferroptotic protection and restoration of the Nrf2/SLC7A11/GPX4 signaling pathway. (**A**) Effects of ML385 (1 μM) on baicalein-mediated rescue of AC16 cell viability (n = 6). (**B**) Effects of ML385 on baicalein-mediated restoration of the GSH/GSSG ratio in HG + PA-treated AC16 cells (n = 6). (**C**) Effects of ML385 on the baicalein-mediated reduction in MDA levels in HG + PA-treated AC16 cells (n = 6). (**D**,**E**) Effects of ML385 on the baicalein-mediated reduction in intracellular Fe^2+^ levels in HG + PA-treated AC16 cells (scale bar = 100 μm) (n = 3). (**F**,**G**) Representative C11-BODIPY 581/591 fluorescence images showing lipid peroxidation in AC16 cells (red, non-oxidized probe; green, oxidized probe; scale bar = 100 μm) (n = 3). (**H**,**I**) Effects of baicalein alone or in combination with ML385 on the relative mRNA expression levels of *ACSL4* and *PTGS2* in HG + PA-treated AC16 cells (n = 6). (**J**–**M**) Effects of baicalein and ML385 on Nrf2/SLC7A11/GPX4 signaling in HG + PA-treated AC16 cells. Data are presented as mean ± SD. * *p* < 0.05, ** *p* < 0.01, *** *p* < 0.001; ns, not significant.

To determine whether Nrf2-dependent regulation of ferroptosis contributes to the protective effects of baicalein under HG + PA stress, AC16 cells were treated with ML385 (1 μM), a pharmacological inhibitor of Nrf2. HG + PA exposure significantly reduced cell viability, whereas baicalein partially restored viability under these conditions. ML385 alone had no significant effect on cell viability in control cells but significantly attenuated the protective effect of baicalein under HG + PA stress, as indicated by lower viability in the HG + PA + Bai + ML385 group than in the HG + PA + Bai group (Figure 5A).

We then examined ferroptosis-related biochemical alterations. HG + PA exposure decreased the GSH/GSSG ratio and increased MDA levels and FerroOrange fluorescence intensity, indicating disrupted glutathione redox homeostasis, enhanced lipid peroxidation, and intracellular labile Fe^2+^ accumulation. Baicalein markedly ameliorated these changes, whereas ML385 diminished its effects, resulting in a lower GSH/GSSG ratio and higher MDA levels and FerroOrange fluorescence intensity (Figure 5B–E). In parallel, C11-BODIPY 581/591 staining showed that HG + PA increased lipid peroxidation, as reflected by enhanced green fluorescence and reduced red fluorescence. Baicalein attenuated this fluorescence shift, whereas ML385 partially reversed this effect (Figure 5F,G). Collectively, these results indicate that Nrf2 inhibition weakens the ability of baicalein to suppress HG + PA-induced ferroptosis-related changes.

This pattern was further corroborated by transcriptional analysis. HG + PA exposure significantly increased the mRNA expression of pro-ferroptotic genes *ACSL4* and *PTGS2*, two ferroptosis-associated genes, in AC16 cells. Baicalein reduced the expression of both genes, whereas ML385 partially reversed these effects, as shown by higher *ACSL4* and *PTGS2* mRNA levels in the HG + PA + Bai + ML385 group than in the HG + PA + Bai group (Figure 5H,I).

**Figure 6:**
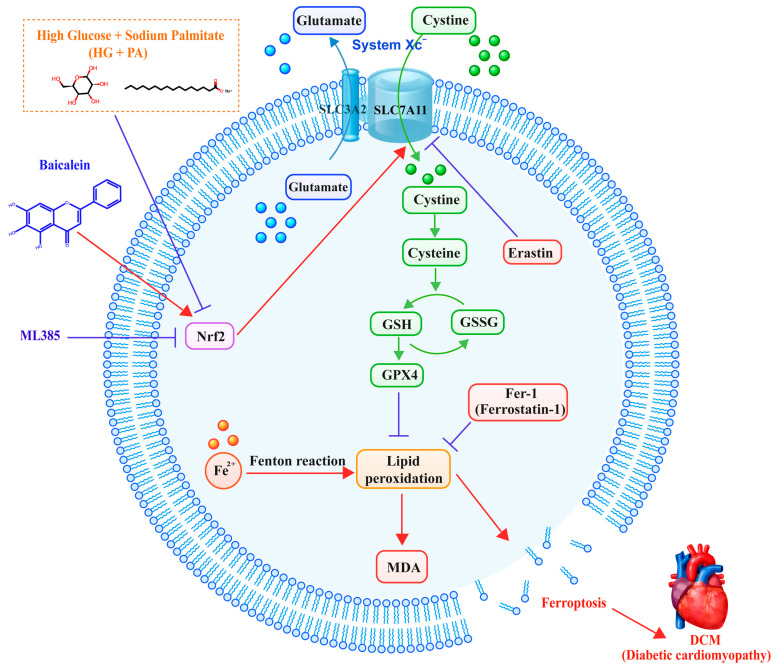
Schematic diagram showing the protective mechanism of baicalein against HG + PA-induced ferroptosis in cardiomyocytes.

Consistent with these changes, Western blot analysis showed that HG + PA exposure reduced the protein levels of Nrf2, SLC7A11, and GPX4 in AC16 cells. Baicalein partially restored the protein abundance of Nrf2, SLC7A11, and GPX4 under HG + PA conditions, whereas ML385 attenuated this response (Figure 5J–M). Taken together, these findings suggest that baicalein protects AC16 cells against HG + PA-induced ferroptosis-related injury, at least in part, through activation of the Nrf2/SLC7A11/GPX4 axis, and that pharmacological inhibition of Nrf2 compromises this protection. A schematic overview of the proposed mechanism is presented in Figure 6.

## 3. Discussion

Diabetic cardiomyopathy (DCM) is a major cardiac complication of diabetes mellitus (DM) and contributes to adverse cardiac remodeling and heart failure [51]. Metabolic disturbances, including insulin resistance, compensatory hyperinsulinemia, and persistent hyperglycemia, are central to its development [52]. DCM is generally characterized by myocardial dysfunction that cannot be fully explained by conventional cardiac risk factors such as coronary artery disease, valvular heart disease, or hypertension [53]. Increasing evidence indicates that ferroptosis, an iron-dependent form of regulated cell death driven by lipid peroxidation, participates in the pathogenesis of DCM [54]. Baicalein has been reported to exert anti-ferroptotic activity in several disease models [55,56] and to protect cardiomyocytes under diabetic stress [35]. In this study, we found that baicalein attenuated high glucose + sodium palmitate (HG + PA)-induced cardiomyocyte injury, and this effect was closely associated with suppression of ferroptosis and restoration of the Nrf2/SLC7A11/GPX4 axis.

We first established HG + PA-induced injury models in H9c2 and AC16 cells to mimic metabolic stress relevant to DCM. Baicalein improved cell viability, reduced cell death, and decreased LDH release in both cell models, indicating a reproducible cytoprotective effect against HG + PA-induced injury. The effective concentrations differed between the two cell lines, which may reflect species- and cell type-dependent differences in basal metabolic characteristics, stress susceptibility, and pharmacological responsiveness. Since small-molecule studies targeting ferroptosis often validate pharmacological activity across more than one experimental model before mechanistic investigation [57], the protective phenotype observed in both cell types strengthens the reliability of the initial screening results. Subsequent mechanistic experiments were primarily conducted in AC16 cells because their derivation from adult human ventricular tissue provides a human-relevant and well-established cellular model for investigating cardiomyocyte responses to diabetic metabolic stress [58,59,60].

Integrated network pharmacology, transcriptomic analysis, and pathway enrichment highlighted ferroptosis as a candidate mechanism involved in baicalein-mediated protection. This prediction was further supported by pharmacological modulation experiments. Fer-1 improved the viability of HG + PA-treated AC16 cells, suggesting that ferroptosis contributes to HG + PA-induced cytotoxicity. Conversely, Erastin partially weakened the viability-restoring effects of baicalein, indicating that ferroptosis activation counteracts baicalein-mediated cytoprotection. These findings support ferroptosis as a functionally relevant component of HG + PA-induced cardiomyocyte injury, although other forms of metabolic stress-related cell damage may also be involved.

Further enrichment and target–pathway analyses suggested that the Nrf2/SLC7A11/GPX4 axis may be an important regulatory pathway involved in this process. The Nrf2/SLC7A11/GPX4 axis is a central antioxidant defense system against ferroptosis [61]. Nrf2 regulates genes involved in oxidative stress response, detoxification, metabolism, inflammation, and ferroptosis-related processes [62]. SLC7A11, a key component of system Xc^−^, promotes cystine uptake and supports glutathione synthesis [63,64,65], whereas GPX4 uses reduced glutathione to detoxify lipid hydroperoxides and limit ferroptotic cell death [66,67,68,69]. Previous studies have shown that Nrf2 signaling can regulate SLC7A11 expression and support the GSH–GPX4 antioxidant system, thereby enhancing cellular resistance to ferroptosis, including in cardiomyocytes [70,71,72,73]. Therefore, the enrichment of oxidative stress, glutathione metabolism, and ferroptosis-related terms in the drug–disease intersecting targets provided a mechanistic basis for focusing on this axis.

Consistent with the pathway–target analysis, molecular docking and molecular dynamics simulations provided computational support for the potential involvement of the Nrf2/SLC7A11/GPX4 axis in baicalein-mediated ferroptosis regulation. Molecular docking predicted favorable interactions between baicalein and Nrf2, SLC7A11 and GPX4, while 100-ns molecular dynamics simulations further suggested that the predicted baicalein–target complexes were generally stable. These results supported the selection of the Nrf2/SLC7A11/GPX4 axis for subsequent functional validation. However, docking and molecular dynamics simulations are predictive approaches and should be interpreted as supportive evidence rather than direct proof of physical binding.

To further assess the contribution of Nrf2 signaling to the anti-ferroptotic effects of baicalein, we used ML385, a pharmacological inhibitor that suppresses Nrf2-dependent transcription by interfering with the binding of Nrf2–MafG complexes to antioxidant response elements [74]. In the present study, ML385 partially attenuated the effects of baicalein on HG + PA-induced oxidative and ferroptosis-associated abnormalities, as evidenced by the rebound in *ACSL4* and *PTGS2* expression, lipid peroxidation, and intracellular labile Fe^2+^ accumulation. *ACSL4* promotes the incorporation of polyunsaturated fatty acids into membrane phospholipids, thereby increasing the pool of oxidizable lipid substrates and cellular susceptibility to ferroptosis, whereas *PTGS2* is frequently used as a ferroptosis-associated transcriptional indicator. Thus, changes in *ACSL4* and *PTGS2* provide complementary support for the involvement of ferroptosis-associated processes in HG + PA-induced injury by reflecting alterations in membrane lipid remodeling and ferroptosis-related transcriptional responses, respectively. In parallel, ML385 partially blunted the baicalein-mediated restoration of Nrf2, SLC7A11, and GPX4 protein levels under HG + PA stress. These consistent molecular and biochemical changes suggest that restoration of the Nrf2/SLC7A11/GPX4 axis contributes to the ability of baicalein to alleviate ferroptosis-associated cardiomyocyte injury. Importantly, ML385 did not completely abolish the protective effects of baicalein, indicating that Nrf2 signaling plays an important, but not exclusive, role in baicalein-mediated protection. Additional Nrf2-independent processes involving iron homeostasis, membrane lipid remodeling, or other antioxidant pathways may also contribute to the overall protective effects of baicalein and warrant further investigation.

Several limitations should be acknowledged. First, the present study was conducted using cultured cardiomyocyte models. Although HG + PA exposure reproduces selected features of glucolipotoxic stress associated with diabetes, it cannot fully recapitulate the complex metabolic, inflammatory, neurohumoral, vascular, and hemodynamic environment of diabetic cardiomyopathy in vivo. Therefore, the current findings support the protective effects of baicalein at the cellular level but do not establish whether similar effects occur in the diabetic heart in vivo. Further studies using appropriate animal models of diabetic cardiomyopathy are required to evaluate the effects of baicalein on cardiac function, cardiac remodeling, and histopathological alterations and to determine whether these effects are accompanied by modulation of ferroptosis-related processes and the Nrf2/SLC7A11/GPX4 axis.

Second, the mechanistic evidence for Nrf2 involvement was based primarily on pharmacological inhibition with ML385. Although the partial reversal observed following ML385 treatment supports the contribution of Nrf2 signaling to baicalein-mediated protection, pharmacological inhibitors may exert concentration-dependent, context-dependent, or off-target effects. Thus, the present results are insufficient to establish whether Nrf2 is required for this protective effect. Future studies using Nrf2 knockdown or knockout, complemented by Nrf2 overexpression and re-expression rescue experiments, are warranted to provide more definitive evidence of pathway dependence. Further manipulation of SLC7A11 or GPX4 may also help clarify the relative contributions of individual components of this axis.

Third, molecular docking and molecular dynamics simulations provide computational predictions of potential binding modes but do not establish direct molecular binding or cellular target engagement. The predicted binding poses, estimated binding energies, and apparent stability of the modeled complexes during simulation generate testable computational hypotheses regarding whether baicalein could interact with Nrf2, SLC7A11, or GPX4 under experimental conditions. These findings should therefore be regarded as hypothesis-generating rather than confirmatory. Direct biochemical, biophysical, or cellular target-engagement approaches, such as surface plasmon resonance, isothermal titration calorimetry, or cellular thermal shift assays, are required to determine whether baicalein directly binds to these proteins or instead modulates the pathway indirectly through upstream regulatory mechanisms.

Finally, HG + PA-induced cardiomyocyte injury involves multiple interconnected forms of metabolic and oxidative stress. Although findings from pharmacological interventions and biochemical and molecular analyses support the involvement of ferroptosis-associated mechanisms, further studies are required to examine the interactions of ferroptosis with other stress-response pathways and other forms of regulated cell death under diabetic conditions.

In conclusion, the present study suggests that baicalein attenuates HG + PA-induced glucolipotoxic injury in cardiomyocyte models. Its protective effects are closely associated with reductions in oxidative damage, lipid peroxidation, and intracellular labile Fe^2+^ accumulation, together with restoration of the Nrf2/SLC7A11/GPX4 axis. The partial reversal of these effects by ML385 indicates that Nrf2-dependent signaling plays an important, but not exclusive, role in baicalein-mediated protection. The consistent changes in several ferroptosis-related indicators further support the involvement of ferroptosis-associated processes in baicalein-mediated protection. Computational analyses generate testable structural hypotheses regarding possible interactions between baicalein and Nrf2, SLC7A11, or GPX4 but should not be interpreted as evidence of direct target binding. Collectively, these findings provide mechanistic support for further investigation of the protective potential of baicalein against cardiomyocyte injury associated with diabetic metabolic stress. Further in vivo studies are required to evaluate its therapeutic potential, and genetic loss- and gain-of-function experiments, together with target-engagement analyses, are needed to clarify its pathway dependence and potential direct molecular targets.

## 4. Materials and Methods

### 4.1. Cell Culture

Cells were cultured in a humidified incubator (Heracell VIOS 160i; Thermo Fisher Scientific, Waltham, MA, USA) at 37 °C with 5% CO_2_. The human AC16 cardiomyocyte cell line (Merck Millipore, Burlington, MA, USA; Cat. No. SCC109) was maintained in complete culture medium consisting of Corning^®^ high-glucose Dulbecco’s modified Eagle’s medium (DMEM; Corning, NY, USA; Cat. No. 10-013-CV), supplemented with 10% fetal bovine serum (FBS; Gibco, Grand Island, NY, USA; Cat. No. 10091148) and 1% penicillin–streptomycin (Gibco, Grand Island, NY, USA; Cat. No. 15140122). The rat H9c2 cardiomyoblast cell line (Cell Bank of the Chinese Academy of Sciences, Shanghai, China; Cat. No. GNR5) was cultured in complete medium prepared using DMEM containing 1.5 g/L NaHCO_3_ (iCell Bioscience Inc., Shanghai, China; Cat. No. iCell-128-0001), supplemented with 10% FBS and 1% penicillin–streptomycin. When the cells reached approximately 85% confluence, they were washed twice with phosphate-buffered saline (PBS; Procell Life Science & Technology Co., Ltd., Wuhan, China; Cat. No. PB180327), digested with 0.25% trypsin (Gibco, Thermo Fisher Scientific, Grand Island, NY, USA; Cat. No. 15050065), and subsequently passaged or seeded for further experiments.

### 4.2. Cell Viability Assay

To determine the appropriate concentration for model induction, cell viability was assessed using the Cell Counting Kit-8 (CCK-8; Beyotime Biotechnology, Shanghai, China; Cat. No. C0038). Cultured H9c2 and AC16 cells were digested with trypsin, centrifuged, and resuspended before being seeded into 96-well plates (Corning, Corning, NY, USA; Cat. No. 3599). After 24 h of incubation, different treatments were administered. The model was established by supplementing the culture medium with D-(+)-glucose (Sangon Biotech Co., Ltd., Shanghai, China; Cat. No. A501991-0500) to achieve final glucose concentrations of 33 mmol/L for H9c2 cells [75] and 45 mmol/L for AC16 cells [76], together with various concentrations of sodium palmitate (PA; Xi’an Kunchuang Science and Technology Development Co., Ltd., Xi’an, China; Cat. No. SYSJ-KJ002). During the determination of the PA IC_50_, a corresponding vehicle control was included. Following 24 h of drug treatment, 10% CCK-8 solution was added to each well, and the plates were incubated for 2 h. Absorbance was then measured at 450 nm using a microplate reader (Synergy 2; BioTek Instruments, Winooski, VT, USA). Cell viability was calculated using the formula: [(A_drug_ − A_blank_)/(Ac_ontrol_ − A_blank_) × 100%], and the IC_50_ of PA was determined using GraphPad software (10.1.2).

Using the IC_50_-derived concentration of PA, the optimal protective concentration of baicalein (Tauto Biotech Co., Ltd., Shanghai, China; Cat. No. E-0051; purity ≥ 98%) was determined. H9c2 and AC16 cells were treated with HG + PA in the presence or absence of various concentrations of baicalein for 24 h. Cell viability was subsequently assessed using the CCK-8 assay, and dose–response curves were generated using GraphPad Prism software (version 10.1.2; GraphPad Software, Boston, MA, USA). For ferroptosis intervention experiments, AC16 cells were pretreated with Erastin (MedChemExpress, Shanghai, China; Cat. No. HY-15763; 2 μmol/L) or ferrostatin-1 (Fer-1; MedChemExpress, Shanghai, China; Cat. No. HY-100579; 3 μmol/L) for 2 h before HG + PA treatment [77]. To evaluate the involvement of Nrf2 signaling in the protective effects of baicalein, AC16 cells were treated with the Nrf2 inhibitor ML385 (MedChemExpress, Shanghai, China; Cat. No. HY-100523) at a final concentration of 1 μmol/L [78]. For the ML385-related cell viability assay, cells were assigned to six groups: Control, HG + PA, HG + PA + Bai, HG + PA + Bai + ML385, HG + PA + ML385 and Control + ML385. Cell viability was assessed after 24 h of treatment. In all cell experiments conducted in this study, Dimethyl sulfoxide (DMSO; Biosharp, Beijing, China; Cat. No. BL165B) was added as required to ensure an identical final DMSO concentration across all groups, including the control, HG + PA, HG + PA + Bai, and inhibitor-treated groups. The final DMSO concentration was maintained below 0.1% (*v*/*v*).

### 4.3. Calcein-AM/Propidium Iodide (PI) Staining

After the culture medium was removed, 1 mL of Calcein-AM/PI double-staining (Dojindo Laboratories, Kumamoto, Japan; Cat. No. C542) working solution, prepared by adding 10 μL of Calcein-AM and 15 μL of PI to 5 mL of PBS, was added to each well. The cells were incubated in the dark for 30 min [79], after which fluorescence images were acquired using a fluorescence microscope (BZ-X810, KEYENCE, Osaka, Japan). Viable cells were identified by green Calcein-AM fluorescence, whereas dead cells were identified by red PI fluorescence. Calcein-AM-positive and PI-positive cells were quantified using Fiji (ImageJ 1.54p). Cell death was expressed as the ratio of PI-positive cells to Calcein-AM-positive cells and normalized to the mean value of the Control group.

### 4.4. Lactate Dehydrogenase (LDH) Cytotoxicity Assay

Exponentially growing AC16 and H9c2 cells were seeded into 100 mm tissue culture-treated dishes (Corning, Corning, NY, USA; Cat. No. 430167). After 24 h, once the cells had adhered, the indicated treatments were applied. Following 24 h of treatment, the culture supernatants from each group were collected, and LDH levels were measured using an LDH assay kit (Beyotime Biotechnology, Shanghai, China; Cat. No. C0016) according to the manufacturer’s instructions [80].

### 4.5. Network Pharmacology

To explore the mechanisms underlying the therapeutic effects of baicalein on DCM, a network pharmacology approach was employed. The structural file of baicalein was obtained from PubChem (https://pubchem.ncbi.nlm.nih.gov/; accessed on 7 June 2026). The potential targets of baicalein were identified using databases such as BATMAN-TCM (https://bionet.ncpsb.org.cn/batman-tcm/; accessed on 7 June 2026), SwissTargetPrediction (http://www.swisstargetprediction.ch/; accessed on 7 June 2026), SuperPred (https://prediction.charite.de/; accessed on 7 June 2026), SEA (https://sea.bkslab.org/; accessed on 7 June 2026), and PharmMapper (https://www.lilab-ecust.cn/pharmmapper/; accessed on 7 June 2026). Relevant target genes associated with DCM were searched using OMIM (https://omim.org/; accessed on 7 June 2026), GeneCards (https://www.genecards.org/; accessed on 7 June 2026), and CTD databases (https://ctdbase.org/; accessed on 7 June 2026). A protein–protein interaction (PPI) network was constructed and visualized using the STRING database (https://string-db.org/; accessed on 7 June 2026) and Cytoscape software (3.10.4; https://cytoscape.org/; accessed on 7 June 2026). GO functional analysis and KEGG enrichment analysis were performed using SRplot (https://www.bioinformatics.com.cn/; accessed on 7 June 2026).

### 4.6. Molecular Docking and Molecular Dynamics Simulations

The three-dimensional structures of the target proteins were obtained from the RCSB Protein Data Bank (https://www.rcsb.org/; accessed on 7 June 2026) or the AlphaFold Protein Structure Database (https://alphafold.ebi.ac.uk/; accessed on 7 June 2026). Specifically, the Nrf2 structure was obtained from AlphaFold DB with the accession number AF-Q16236-F1, whereas the structures of SLC7A11 and GPX4 were retrieved from the PDB database (https://www.rcsb.org/; accessed on 7 June 2026) under the IDs 7P9U and 6ELW, respectively. AutoDock Vina (1.2.3) was employed to perform molecular docking analysis and to estimate the binding affinities between baicalein and the selected protein targets. The most favorable docking conformations were further examined and visualized using PyMOL (3.1.6), and the corresponding ligand–protein interaction patterns were analyzed using LigPlot^+^ (2.3.1).

To validate the reliability of the docking poses generated by AutoDock Vina (1.2.3), the ligand was first separated from the receptor and subjected to redocking. Molecular dynamics (MD) simulations of the protein–ligand complexes were then performed using GROMACS (2025.0; https://www.gromacs.org/; accessed on 7 June 2026). The systems were described with the CHARMM36 force field, solvated using the TIP3P water model in a cubic box with a minimum distance of 1.0 nm from the solute, and neutralized by adding Na^+^ and Cl^−^ ions. After energy minimization using the steepest descent and conjugate gradient algorithms, the systems were equilibrated under NVT conditions at 300 K, followed by NPT equilibration at 300 K and 1 bar. Subsequently, 100-ns production simulations were conducted with a time step of 2 fs. Long-range electrostatic interactions were treated using the particle mesh Ewald (PME) method, and periodic boundary conditions were applied in all three dimensions.

Trajectory analyses were performed to evaluate complex stability and interaction characteristics, including protein backbone RMSD, complex RMSD, RMSF, radius of gyration (Rg), solvent-accessible surface area (SASA), and time-dependent protein–ligand hydrogen bonding. Principal component analysis (PCA) was further conducted, and the free energy landscape (FEL) was constructed based on the first two principal components to characterize conformational space sampling.

### 4.7. Intracellular Fe^2+^ Assay

Intracellular Fe^2+^ levels were detected using FerroOrange (Dojindo Laboratories, Kumamoto, Japan; Cat. No. F374). After treatment, the cells were washed three times with HBSS and incubated with 1 μmol/L FerroOrange working solution prepared in HBSS (Servicebio, Wuhan, China; Cat. No. G4203-500ML) at 37 °C in a humidified 5% CO_2_ incubator for 30 min. After staining, the cells were immediately observed without further washing, and fluorescence images were acquired using a fluorescence microscope (BZ-X810, KEYENCE, Osaka, Japan). Fluorescence intensity was quantified using Fiji (ImageJ 1.54p) and normalized to the mean value of the Control group [81].

### 4.8. Malondialdehyde (MDA) Assay

After treatment, the culture medium was discarded, and the cells were washed twice with cold PBS. The cells were then scraped, transferred into 1.5 mL centrifuge tubes, and processed according to the manufacturer’s instructions for the Malondialdehyde (MDA) Assay Kit (Nanjing Jiancheng Bioengineering Institute, Nanjing, China; Cat. No. A003-4-1). Briefly, the cell samples were mixed with the assay reagents and vortexed thoroughly. The reaction mixtures were incubated in a 95 °C water bath for 40 min and then cooled under running water. After centrifugation at 4000× *g* for 10 min at 4 °C, the supernatants were collected, and the absorbance was measured at 532 nm using a microplate reader. MDA levels were normalized to the total protein concentration determined by the BCA assay (Beyotime Biotechnology, Shanghai, China; Cat. No. P0010S) and expressed as nmol/mg protein [82].

MDA content was calculated using the following formula: MDA content (nmol/mg protein) = [(A_sample_ − A_blank_)/(A_standard_ − A_blank_)] × C_standard_/sample protein concentration where A_sample_, A_blank_, and A_standard_ represent the absorbance values of the sample, blank, and standard, respectively; C_standard_ was 10 nmol/mL.

### 4.9. GSH (Glutathione)/Glutathione Disulfide (GSSG) Measurement

After 24 h of treatment, cells were collected and processed according to the manufacturer’s instructions for the GSSG/GSH Quantification Kit II (Beyotime Biotechnology, Shanghai, China; Cat. No. S0053). Briefly, the culture medium was discarded, and the cells were washed with cold PBS. Fresh cell samples were lysed with protein removal reagent M solution and centrifuged at 10,000× *g* for 10 min at 4 °C (5424 R; Eppendorf SE, Hamburg, Germany). The supernatants were collected for the measurement of total glutathione and GSSG. After the enzymatic reaction, absorbance was measured at 412 nm using a microplate reader. The concentrations of total glutathione and GSSG were calculated according to the standard curves [83]. The GSH concentration and GSH/GSSG ratio were calculated using the following formulas: GSH = total glutathione − 2 × GSSG; GSH/GSSG ratio = GSH/GSSG.

### 4.10. Western Blotting

For Western blot analysis, AC16 cells were seeded in 100 mm tissue culture-treated dishes and assigned to four groups: Control, HG + PA, HG + PA + Bai, and HG + PA + Bai + ML385. After 24 h of treatment, the cells were lysed with RIPA lysis buffer (Beyotime Biotechnology, Shanghai, China; Cat. No. P0013B), scraped from the dishes, and incubated on ice for 20 min. The lysates were centrifuged at 12,000 rpm for 30 min, and the supernatants were collected for subsequent protein analysis. A prestained protein molecular weight marker (ColorMixed Protein Marker 180, 10–180 kDa; ABclonal, Wuhan, China; Cat. No. RM19001) was used as a molecular weight reference. Following electrophoretic separation (Mini-PROTEAN Tetra Cell; Bio-Rad Laboratories, Hercules, CA, USA) and membrane transfer (Mini Trans-Blot Cell; Bio-Rad Laboratories, Hercules, CA, USA), the membranes were blocked with 5% non-fat milk at room temperature for 1.5 h and subsequently incubated overnight at 4 °C with primary antibodies against Nrf2 (Cell Signaling Technology, Shanghai, China; Cat. No. 20733S), SLC7A11 (ABclonal, Wuhan, China; Cat. No. A13685), GPX4 (ABclonal, Wuhan, China; Cat. No. A11243), and β-actin (Cell Signaling Technology, Shanghai, China; Cat. No. 4967S), each diluted at 1:1000. The following day, the membranes were washed three times with 1× TBST buffer (Servicebio, Wuhan, China; Cat. No. G2150-1L) for 5 min each and then incubated with an HRP-conjugated anti-rabbit IgG secondary antibody (Cell Signaling Technology, Shanghai, China; Cat. No. 7074; 1:3000) at room temperature for 1.5 h. After three additional washes with TBST for 5 min each, the membranes were incubated with an enhanced chemiluminescence reagent (Beyotime Biotechnology, Shanghai, China; Cat. No. P0018S) for 1 min, and protein bands were visualized using a chemiluminescence imaging system (ChemiDoc; Bio-Rad Laboratories, Hercules, CA, USA) [84]. Band intensities were quantified using Fiji (ImageJ 1.54p) and normalized to β-actin. Statistical analyses were performed using GraphPad Prism (10.1.2).

### 4.11. C11-BODIPY Lipid Peroxidation Assay

Intracellular lipid peroxidation was assessed using a Lipid Peroxidation Probe -BDP 581/591 C11 (Dojindo Laboratories, Kumamoto, Japan, code: L267) according to the manufacturer’s instructions. AC16 cells were seeded in 24-well cell culture plates (Corning Costar, Corning, NY, USA; Cat. No. 3524) and subjected to the indicated treatments. After treatment, the cells were incubated with the C11-BODIPY probe diluted 1:1000 in culture medium at 37 °C in the dark [85]. The cells were then washed with phosphate-buffered saline (PBS) to remove excess probe. Fluorescence images were acquired immediately using an all-in-one fluorescence microscope (BZ-X810, KEYENCE, Osaka, Japan) with a 20× objective under identical imaging settings for all experimental groups. The non-oxidized form of the probe was detected in the red fluorescence channel, whereas the oxidized form was detected in the green fluorescence channel. Fluorescence intensities were quantified using Fiji (ImageJ 1.54p), and lipid peroxidation was expressed as the oxidized/non-oxidized fluorescence ratio, calculated as the green-to-red fluorescence intensity ratio.

### 4.12. RNA Extraction and Reverse Transcription–Quantitative PCR (RT-qPCR)

Total RNA was extracted from AC16 cells using NucleoZOL reagent (MACHEREY-NAGEL, Düren, Germany; Cat. No. 740404.200) according to the manufacturer’s instructions. RNA concentration and purity were determined using a NanoDrop OneC Microvolume UV–Vis Spectrophotometer (Thermo Fisher Scientific, Waltham, MA, USA).

Complementary DNA (cDNA) was synthesized from 1 μg of total RNA using the Evo M-MLV RT Mix Kit with gDNA Clean for qPCR Ver. 2 (Accurate Biotechnology, Changsha, China; Cat. No. AG11728) according to the manufacturer’s instructions. Reverse transcription was performed in a total reaction volume of 20 μL at 37 °C for 15 min, followed by reverse transcriptase inactivation at 85 °C for 5 s. The resulting cDNA was stored at −20 °C until further analysis.

Reverse transcription–quantitative PCR (RT-qPCR) was performed using the SYBR Green Premix Pro Taq HS qPCR Kit (ROX Plus) (Accurate Biotechnology, Changsha, China; Cat. No. AG11718) on a QuantStudio 3 Real-Time PCR System (Applied Biosystems, Thermo Fisher Scientific, Waltham, MA, USA). Each 20-μL reaction mixture contained 10 μL of 2× SYBR Green Pro Taq HS Premix (ROX Plus), 0.4 μL of forward primer (10 μM), 0.4 μL of reverse primer (10 μM), 2 μL of cDNA template, and 7.2 μL of RNase-free water. The amplification conditions were as follows: initial denaturation at 95 °C for 30 s, followed by 40 cycles of denaturation at 95 °C for 5 s and annealing/extension at 60 °C for 30 s. Melting-curve analysis was subsequently performed to verify the specificity of the amplified products [86]. Each biological sample was analyzed in technical triplicate.

The relative mRNA expression levels of *ACSL4* and *PTGS2* were normalized to ACTB as the reference gene and calculated using the 2^−ΔΔCq method. The primer sequences were as follows: *ACSL4* (forward, 5′-CATCCCTGGAGCAGATACTCT-3′; reverse, 5′-TCACTTAGGATTTCCCTGGTCC-3′) [87], *PTGS2* (forward, 5′-CGGTGAAACTCTGGCTAGACAG-3′; reverse, 5′-GCAAACCGTAGATGCTCAGGGA-3′) [88], and *ACTB* (forward, 5′-AGCCTCGCCTTTGCCGATCC-3′; reverse, 5′-ACATGCCGGAGCCGTTGTCG-3′) [89]. All primers were synthesized by Tsingke Biotechnology Co., Ltd., Shanghai, China. Each sample was analyzed in technical triplicate, and the reported data represent six independent biological replicates (n = 6).

### 4.13. Statistical Analysis

All experiments were independently repeated at least three times. Image-based quantification was performed using Fiji software (ImageJ 1.54p), and the schematic diagram was created using Adobe Illustrator 2025 (version 29.3.1). Statistical analyses were performed using GraphPad Prism software (version 10.1.2). Quantitative data are presented as the mean ± standard deviation (mean ± SD). For comparisons among multiple groups, one-way analysis of variance (ANOVA), followed by Tukey’s post hoc test or Sidak’s multiple-comparisons test, was used as appropriate. A value of *p* < 0.05 was considered statistically significant. * *p* < 0.05, ** *p* < 0.01, and *** *p* < 0.001; ns, not significant.

## Data Availability

The original contributions presented in this study are included in the article/Supplementary Materials. Further inquiries can be directed to the corresponding authors.

## Supplementary Materials

The following supporting information can be downloaded at: https://www.mdpi.com/article/10.3390/ijms27146391/s1.

## Abbreviations

The following abbreviations are used in this manuscript:

DCM	Diabetic cardiomyopathy
DM	Diabetes mellitus
HG	High glucose
PA	Sodium palmitate
LDH	Lactate dehydrogenase
Nrf2/NFE2L2	Nuclear factor erythroid 2-related factor 2
SLC7A11	Solute carrier family 7 member 11
GPX4	Glutathione peroxidase 4
System Xc^−^	Cystine/glutamate antiporter system
GSH	Reduced glutathione
GSSG	Oxidized glutathione
MDA	Malondialdehyde
MafG	Musculoaponeurotic fibrosarcoma oncogene homolog G
ARE	Antioxidant response element
Fer-1	Ferrostatin-1
ROS	Reactive oxygen species
CCK-8	Cell Counting Kit-8
DAPI	4′,6-diamidino-2-phenylindole
MD	Molecular dynamics
GO	Gene Ontology
KEGG	Kyoto Encyclopedia of Genes and Genomes
PPI	Protein–protein interaction
DMSO	Dimethyl sulfoxide
SD	Standard deviation
BSA	Bovine serum albumin
*ACSL4*	acyl-CoA synthetase long-chain family member 4
*PTGS2*	prostaglandin-endoperoxide synthase 2
qPCR	quantitative real-time polymerase chain reaction
ACTB	actin beta
Calcein-AM	calcein acetoxymethyl ester
PI	Propidium iodide
TBST	Tris-buffered saline with Tween 20
HRP	horseradish peroxidase
WB	Western blotting
